# Disparities in Hepatocellular Carcinoma Survival by Insurance Status: A Population-Based Study in China

**DOI:** 10.3389/fpubh.2021.742355

**Published:** 2021-11-05

**Authors:** Jing Wu, Chengyu Liu, Fengmei Wang

**Affiliations:** ^1^School of Pharmaceutical Science and Technology, Tianjin University, Tianjin, China; ^2^Center for Social Science Survey and Data, Tianjin University, Tianjin, China; ^3^The Department of Gastroenterology and Hepatology, Tianjin Third Central Hospital, Tianjin, China

**Keywords:** hepatocellular carcinoma, survival, health disparities, insurance, China

## Abstract

**Objective:** Health disparities related to basic medical insurance in China have not been sufficiently examined, particularly among patients with hepatocellular carcinoma (HCC). This study aims to investigate the disparities in HCC survival by insurance status in Tianjin, China.

**Methods:** This retrospective analysis used data from the Tianjin Basic Medical Insurance claims database, which consists of enrollees covered by Urban Employee Basic Medical Insurance (UEBMI) and Urban and Rural Resident Basic Medical Insurance (URRBMI). Adult patients newly diagnosed with HCC between 2011 and 2016 were identified and followed until death from any cause, withdrawal from UEBMI or URRBMI, or the latest data in the dataset (censoring as of December 31st 2017), whichever occurred first. Patients' overall survival during the follow-up was assessed using Kaplan-Meier and extrapolated by six parametric models. The hazard ratio (HR) and 95% confidence intervals (CI) were calculated with the adjusted Cox proportional hazards model including age at diagnosis, sex, baseline comorbidities and complications, baseline healthcare resources utilization and medical costs, tumor metastasis at diagnosis, the initial treatment after diagnosis and antiviral therapy during the follow-up.

**Results:** Two thousand sixty eight patients covered by UEBMI (*N* = 1,468) and URRBMI (*N* = 570) were included (mean age: 60.6 vs. 60.9, *p* = 0.667; female: 31.8 vs. 27.7%, *p* = 0.074). The median survival time for patients within the UEBMI and URRBMI were 37.8 and 12.2 months, and the 1-, 3-, 5-, 10-year overall survival rates were 63.8, 50.2, 51.0, 33.4, and 44.4, 22.8, 31.5, 13.1%, respectively. Compared with UEBMI, patients covered by URRBMI had 72% (HR: 1.72; 95% CI: 1.47–2.00) higher risk of death after adjustments for measured confounders above. The survival difference was still statistically significant (HR: 1.49; 95% CI: 1.21–1.83) in sensitivity analysis based on propensity score matching.

**Conclusions:** This study reveals that HCC patients covered by URRBMI may have worse survival than patients covered by UEBMI. Further efforts are warranted to understand healthcare disparities for patients covered by different basic medical insurance in China.

## Introduction

Primary liver cancer is the sixth commonly diagnosed cancer and the third leading cause of cancer death worldwide, with about 905,667 new cases and 830,180 deaths in 2020 (1). China is the most afflicted country with almost half of global newly diagnosed patients and fatalities (410,038 new cases and 391,152 deaths in 2020) (2). Moreover, the prognosis for Chinese with primary liver cancer is inferior than other countries and regions, with a 5-year survival probability of only 14.1% (3). Hepatocellular carcinoma (HCC) accounts for ~90% of all local primary liver cancer, followed by intrahepatic cholangiocarcinoma amongst other types (4). Effective HCC treatment options, depending on the tumor stage and the underlying liver function, include hepatectomy, liver transplantation, transarterial chemoembolization (TACE), ablation, radiotherapy, and systemic therapies. Previous studies have indicated that patients with cancer may alter treatment options to reduce the out-of-pocket expenses and ease their financial burden (5).

Health insurance positively affects cancer diagnostics and treatments as it decreases patients' financial burden (6, 7). A previous study reported that patients with no insurance were more likely to be diagnosed at an advanced stage for all cancers when compared to those with private insurance (7). Furthermore, many studies have claimed that insurance status might be an important prognostic factor because of its impact on access to health care (8–18). Two studies have reported that Medicare or commercial insurance, compared with Medicaid or no insurance, were associated with improved HCC survival in the United States (15, 16). This association was declared in several other cancers, such as breast, lung, colorectal, bladder, multiple myeloma, and follicular lymphoma (9–14). However, the relationship between insurance status and cancer survival has not been extensively studied in China.

As the largest developing country, China has launched basic medical insurance schemes in the 1990s. After more than ten years of development, near-universal health insurance coverage was achieved in 2011, which consisted of three schemes: Urban Employee Basic Medical Insurance (UEBMI) for enrollees; Urban Resident Basic Medical Insurance (URBMI) for children, students and other unemployed adult residents living in urban areas; and New Rural Cooperative Medical Scheme (NRCMS) for all residents living in rural areas (19, 20). As these three insurance schemes were initially designed for individuals with different affordability of healthcare services based on their financial situation, benefits packages were quite different. Compared with UEBMI, patients enrolled in URBMI or NRCMS were underinsured which meant that they had lower reimbursement rate and limited coverage (20). In 2016, the URBMI and NRCMS were merged to form the Urban and Rural Resident Basic Medical Insurance (URRBMI) to improve administrative efficiency (19). URRBMI and UEBMI covered 13.61 billion inhabitants accounting for 96.4% of the total Chinese population in 2020 (21). However, the differences in benefits packages still exist between the current two basic medical insurances in China (21).

So far, only two studies have reported the disparities in cancer survival related to basic medical insurance in China (22, 23). One study revealed that non-small cell lung cancer patients enrolled in insurance plans with higher reimbursement rate or broader coverage (UEBMI or Free Medical Care) had better survival rates than those with inadequate insurance (uninsured or NRCMS) (22). The other study suggested that underinsured patients (NRCMS) faced a higher risk of breast cancer-specific mortality (23). However, the relationship between basic medical insurance and HCC survival was not reported.

Tianjin, one of the four municipalities in China, is the largest coastal city located in the Northern part of mainland China, and ranks 7th among all 31 provinces/municipalities regarding Gross Domestic Product per capita (GDP). In addition, Tianjin is the first provincial-level region that have achieved the integration of URBMI and NRCMS schemes in China and has established a relatively comprehensive basic medical insurance system. By 2020, there were about 11.64 million enrollees (UEBMI: 6.18 million, URRBMI: 5.46 million) in the northern municipality (24). This study aims to investigate the disparities in HCC survival by insurance status in Tianjin, China.

## Materials and Methods

### Data Source

This population-based study was conducted on data obtained from the Tianjin Basic Medical Insurance claims database (2008–2017), which consists of enrollees covered by UEBMI and URRBMI. The database consisted of inpatient, outpatient and pharmacy services claims. Enrollment history, patient demographics (age, sex, working status), dates of service, diagnoses, information on medical prescriptions and procedures, and related costs were recorded in this database. International Statistical Classification of Diseases and Related Health Problems 10th Revision (ICD-10) codes and medical records were used to identify the disease diagnoses. In addition, all-cause mortality information was included in a separate dataset, which could be linked by patients' unique identification number. This study was exempted from applying for ethical approval by the Safety and Ethics Committee of the School of Pharmaceutical Science and Technology, Tianjin University.

### Study Population

Males and females, aged over 18 years, with a first discharge diagnosis or outpatient diagnosis of HCC (defined as ICD-10 code C22.0, supplemented by Chinese descriptions), between January 1st 2011 and December 31st 2016, were eligible for inclusion. According to the insurance at the time of diagnosis, patients were grouped into two categories, UEBMI and URRBMI. The date of the first recorded HCC diagnosis was defined as the index date, and the 12 months before the index date was defined as the baseline period. Patients who were not continuously enrolled in the UEBMI or URRBMI during the 12 months prior to the index date, and patients who had history of any malignant neoplasm during the baseline period, were excluded. The cohort was followed until death from any cause, withdrawal from UEBMI or URRBMI, or the latest data in the dataset (censoring as of December 31st 2017), whichever occurred first. All patients in this study were continuously enrolled in only one type of insurance (UEBMI or URRBMI) during the whole study period, including the baseline and follow-up periods.

### Outcomes Measures

Overall survival, measured in months, was calculated from the index date to the date of death, December 31st 2017, or the last enrollment date, whichever occurred first. Survival for patients still alive at the end of their follow-up period were censored.

### Covariates of Interest

The primary covariate of interest was the insurance status, which was described as UEBMI or URRBMI. Additional covariates of interest included the following: age at diagnosis (categorized as 18–44, 45–54, 55–64, 65–74, or >75 years), sex (male or female), baseline healthcare resources use and medical costs (any hospitalizations, average length of stay per hospitalization, any outpatient visits and total direct medical costs), baseline Charlson Comorbidity Index (CCI) score [computed using an algorithm provided by Quan et al. (25)], liver comorbidities and complications (including hepatitis, liver cirrhosis, fatty liver, alcoholic liver, liver failure, as well as portal hypertension, hepatorenal syndrome, ascites, esophageal variceal bleeding, hepatic encephalopathy, jaundice, and primary peritonitis), tumor status at diagnosis (metastasis or not) and initial treatment after diagnosis which may represented the severity of the disease to some extent and was broadly categorized as curative surgery (including hepatectomy and liver transplantation), non-curative surgery (including TACE and ablation), or no surgery. The ICD-10 codes used for the identification of liver comorbidities and complications were listed were reported in the Supplementary Table S1. In addition, antiviral therapy was considered since it could significantly improve the liver function of HCC patients (26, 27). Patients who had at least two prescriptions of antiviral medication during the follow-up period were defined as receiving antiviral therapy.

### Statistical Analysis

Descriptive statistics were performed to estimate the patients' characteristics for the UEBMI cohort, the URRBMI cohort and all patients. The *t-test* and the chi-squared test were employed for continuous variable and categorical variables, respectively, to determine the significant differences in characteristics between the two cohorts.

Patients' overall survival during the follow-up period was estimated by the Kaplan-Meier method and compared with a log-rank test. The hazard ratio (HR) and 95% confidence intervals (CI) were calculated with adjusted Cox proportional hazards models. Age at diagnosis, sex, baseline healthcare resources utilization and medical costs were adjusted in the Model A. CCI score and baseline liver comorbidities and complications were additionally included in Model B on the basis of Model A. Model C was adjusted for tumor metastasis at diagnosis and initial treatment after diagnosis as the proxy of the severity of HCC on the basis of Model B. Model D was carried out with additional adjustment for antiviral therapy during the follow-up aiming at excluding the effect of non-anticancer therapy on HCC survival. The proportionality hazards assumption was tested by the Schoenfeld residual method. As the initial treatment may violate the proportionality assumption, the Model C and Model D were stratified by initial treatment (28). In addition, the Cox models were also adjusted for the calendar year; however, due to violating the proportionality assumption and the lack of statistically meaningful differences, it had not been included in the final model.

The lifetime survival beyond the follow-up period for the UEBMI cohort, the URRBMI cohort, and all patients were estimated by extrapolating the Kaplan-Meier survival curves. Six distributions for the parameters were considered, including Exponential, Weibull, Gompertz, Log-logistic, Log-normal and Generalized gamma. The lifetime in this study was defined as 100 years old based on the average life expectancy of Tianjin residents (81.79 years old in 2019) (29). The Log-normal model fitted better than other parameter distributions for all cohorts based on the assessment using Akaike's information criterion (AIC), Bayesian information criterion (BIC), and the visual inspection method (Supplementary Table S2, Supplementary Figures S1–S3). As the suboptimal distribution, Generalized gamma models were used for sensitivity analysis (Supplementary Figure S4).

In addition, to minimize potential bias, propensity scores were calculated by multivariate logistic regression including age, sex, CCI score, liver comorbidities and complications, tumor metastasis at diagnosis, as well as baseline healthcare resources utilization and medical costs (Supplementary Table S3). Two matched cohorts were identified using one-to-one nearest neighbor matching without replacement, with a caliper of 0.0008. Sensitivity analysis based on the cohorts after matching was performed to assess the robustness of the results.

The significant level was defined as two-sided alpha = 0.05. All statistical analyses were performed using Stata statistical software (version 13.0; StataCorp, College Station, Texas).

## Results

### Baseline Characteristics

**Figure 1:**
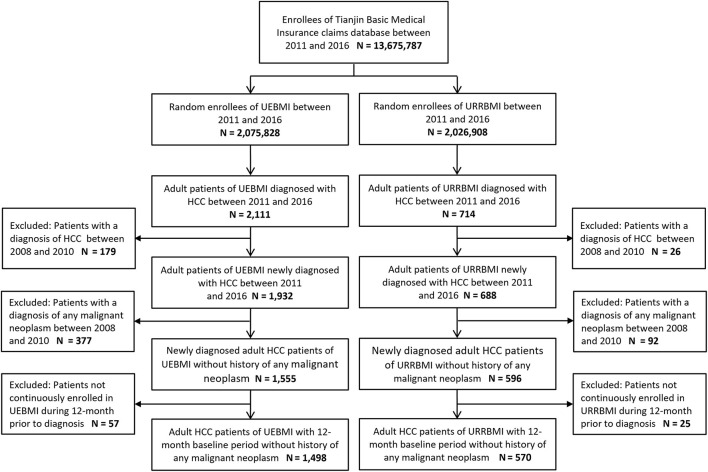
Sample selection flowchart. UEBMI, Urban Employee Basic Medical Insurance; URRBMI, Urban and Rural Resident Basic Medical Insurance; HCC, hepatocellular carcinoma.

A total of 2,068 patients newly diagnosed with HCC were identified, of which UEBMI covered 1,468 and 570 were covered by URRBMI (Figure 1). The mean age of the total cohort was 60.7 years (UEBMI vs. URRBMI: 60.0 vs. 60.9, *p* = 0.667), with 30.7% females (UEBMI vs. URRBMI: 31.8 vs. 27.7%, *p* = 0.074). Compared with patients covered by UEBMI, those in the URRBMI cohort tended to use fewer healthcare resources (including shorter length of stay per hospitalization and fewer outpatient visits) with lower related medical costs during the baseline period and were with lower CCI scores, but were more likely to be diagnosed with severe liver diseases such as decompensated cirrhosis, liver failure and ascites (Table 1).

**Table 1:** Baseline characteristics for patients with HCC.

	**Overall**	**UEBMI**	**URRBMI**	***P***
	**(*N* = 2,068)**	**(*N* = 1,498)**	**(*N* = 570)**	
**Demographic characteristics**				
Age [Mean (SD)]	60.7 (12.6)	60.6 (12.9)	60.9 (11.6)	0.667
Female [*N* (%)]	634 (30.7%)	476 (31.8%)	158 (27.7%)	0.074
**Comorbidities and complications [*****N*** **(%)]**				
CCI score [Mean (SD)]	4.44 (2.16)	4.69 (2.23)	3.78 (1.81)	**<0.001**
**Comorbidities related to the liver**				
Hepatitis	916 (44.3%)	660 (44.1%)	256 (44.9%)	0.727
HBV	741 (35.8%)	525 (35.0%)	216 (37.9%)	0.227
HCV	82 (4.0%)	69 (4.6%)	13 (2.3%)	**0.015**
Cirrhosis of the liver	939 (45.4%)	663 (44.3%)	276 (48.4%)	0.089
Compensated cirrhosis	490 (23.7%)	365 (24.4%)	125 (21.9%)	0.244
Decompensated cirrhosis^†^	449 (21.7%)	298 (19.9%)	151 (26.5%)	**0.001**
Hepatic failure	266 (12.9%)	176 (11.7%)	90 (15.8%)	**0.014**
Fatty liver disease	92 (4.4%)	79 (5.3%)	13 (2.3%)	**0.003**
Alcoholic liver disease^‡^	52 (2.5%)	42 (2.8%)	10 (1.8%)	0.173
Ascites	366 (17.7%)	232 (15.5%)	134 (23.5%)	**<0.001**
Hepatic encephalopathy	202 (9.8%)	151 (10.1%)	51 (8.9%)	0.438
Jaundice	137 (6.6%)	104 (6.9%)	33 (5.8%)	0.346
Portal hypertension	82 (4.0%)	57 (3.8%)	25 (4.4%)	0.545
Esophageal variceal bleeding	54 (2.6%)	38 (2.5%)	16 (2.8%)	0.731
Primary peritonitis	52 (2.5%)	37 (2.5%)	15 (2.6%)	0.834
Hepatorenal syndrome	35 (1.7%)	31 (2.1%)	4 (0.7%)	**0.031**
**All-cause resource utilization and costs**				
Total cost [Mean(SD), CNY]	7,505 (16,870)	8,940 (17,501)	3,733 (14, 435)	**<0.001**
Any hospitalizations [*N* (%)]	428 (20.7%)	314 (21.0%)	114 (20.0%)	0.630
ALOS per hospitalization [Mean(SD)]	12.9 (10.5)	14.0 (11.4)	9.9 (6.6)	**<0.001**
Any outpatient visits [*N* (%)]	1,578 (76.3%)	1,424 (95.1%)	154 (27.0%)	**<0.001**

*CCI score, Charlson Comorbidity Index score; HBV, hepatitis B virus; HCV, hepatitis C virus; CNY, Chinese yuan (year-2017 1 USD = 6.77 CNY); ALOS, Average length of stay*.

† *Patients with liver cirrhosis who had the following symptoms were defined as decompensated liver cirrhosis: ascites; esophageal variceal bleeding; hepatorenal syndrome; portal hypertension; hepatic encephalopathy and jaundice; hepatic encephalopathy and primary peritonitis; jaundice and primary peritonitis*.

‡ *Including alcoholic liver cirrhosis, alcoholic hepatitis, alcoholic fatty liver disease and alcoholic liver failure; hepatitis, liver cirrhosis, fatty liver disease and liver failure in this table only included non-alcoholic disease. Bold values means P < 0.05*.

### Short-Term Survival of HCC Patients

**Figure 2:**
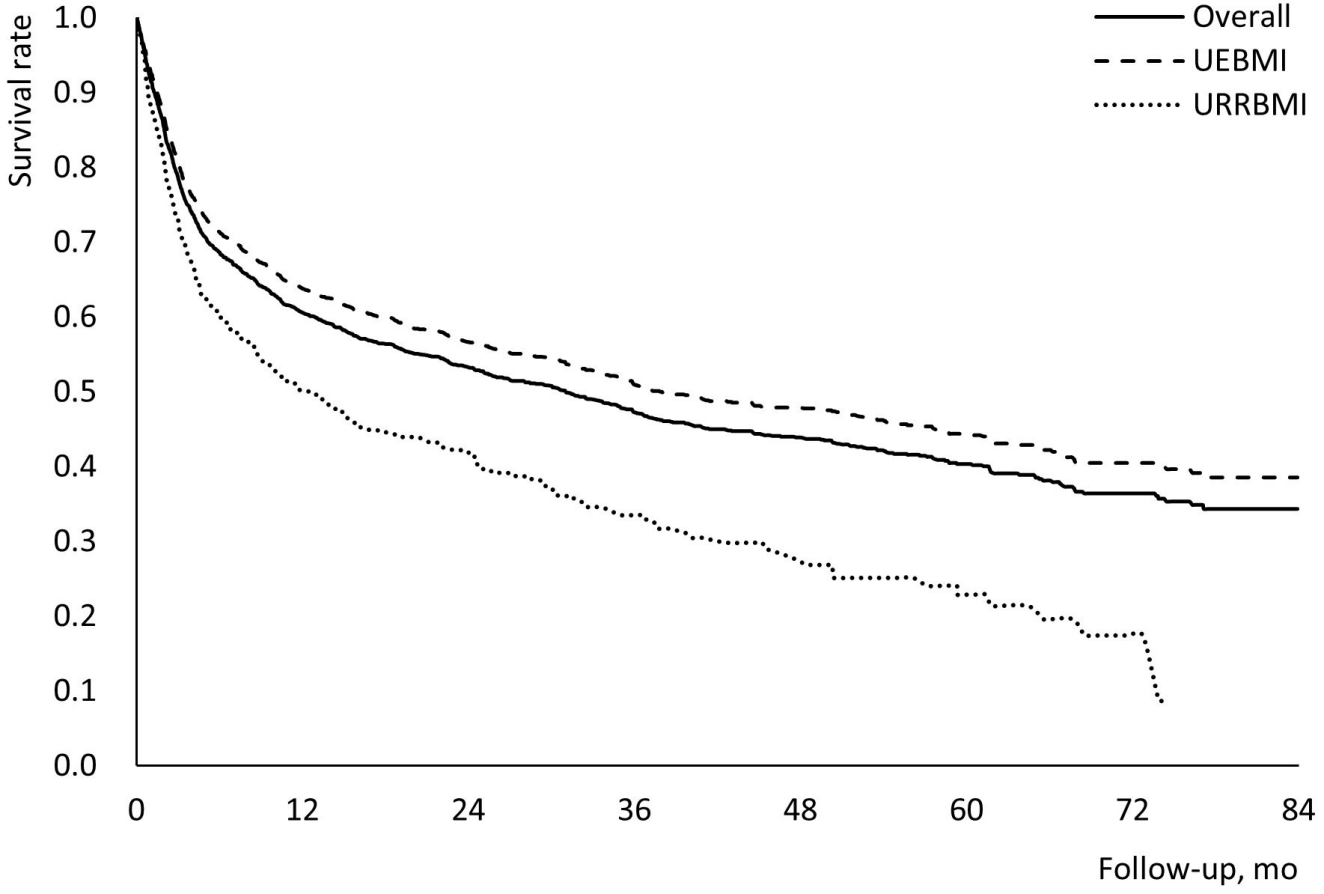
Kaplan-Meier survival curves for patients with HCC during the follow-up period. UEBMI, Urban Employee Basic Medical Insurance; URRBMI, Urban and Rural Resident Basic Medical Insurance; HCC, hepatocellular carcinoma.

During the follow-up period (mean: 25.9 months, median: 16.7 months), 783 and 297 deaths were observed in the UEBMI and the URRBMI cohorts (52.3 vs. 52.1%, *p* = 0.947, Supplementary Table S4). 1-, 3-, 5-year overall survival rates among patients covered by UEBMI were 63.8, 51.0, and 44.4%, compared with 50.2, 33.4, and 22.8% among patients covered by URRBMI (*p* < 0.001; Table 1, Figure 2). There were also statistically significant differences in overall survival among patients in different subgroups (age of 18–44 vs. 45–54 vs. 55–64 vs. 65–74 vs. ≥75; male vs. female; CCI score ≤ 4 vs. CCI score >4; non-cirrhosis vs. compensated cirrhosis vs. decompensated cirrhosis) based on Kaplan-Meier methods and log-rank tests (Supplementary Figure S5). In addition, 1-, 3-, 5-year overall survival rates for all patients with HCC were shown in Table 2.

**Table 2:** Patients' overall survival during the follow-up period.

	**Overall**	**UEBMI**	**URRBMI**
	**(*N* = 2,068)**	**(*N* = 1,498)**	**(*N* = 570)**
**Overall survival, mo**.			
Median	31.0	37.8	12.2
Mean[95%CI]	40.7^*^ [39.0, 42.4]	43.8^*^ [41.9, 45.7]	27.1^*^ [39.0, 42.4]
**Survival rate[95%CI]**			
1-year	60.6 [58.4, 62.7]	63.8 [61.3, 66.2]	50.2 [45.5, 54.8]
3-year	47.3 [44.9, 49.6]	51.0 [48.3, 53.6]	33.4 [28.3, 38.6]
5-year	40.3 [37.8, 42.8]	44.4 [41.5, 47.2]	22.8 [17.1, 29.0]

* *Largest observed analysis time was censored; mean was underestimated*.

In the multiple Cox proportional hazards model that adjusted for measured confounders (Table 3), patients covered by URRBMI had a 72% higher risk of death than those covered by UEBMI (HR: 1.72; 95% CI: 1.47–2.00). Compared with adult patients younger than 45 years old, there was worse survival among patients who were at least 45 years old (45–54 years, HR: 1.79; 95% CI: 1.23–2.59; 55–64 years, HR: 2.45; 95% CI: 1.72–3.49; 65–74 years, HR: 3.43; 95% CI: 2.40–4.89; ≥75 years, HR: 5.25; 95% CI: 3.66–7.53). Moreover, male patients had a higher risk of death than females (HR: 1.74; 95% CI: 1.51–2.00). In addition, some factors also appeared to be associated with decreased or increased survival in the multiple Cox model, including compensated cirrhosis (HR: 1.34; 95% CI: 1.12–1.60), decompensated cirrhosis (HR: 1.85; 95% CI: 1.54–2.21), baseline outpatient visit (HR: 2.01; 95% CI: 1.72–2.34), tumor metastasis (HR: 2.58; 95% CI: 2.19–3.04), fatty liver disease (HR: 0.66; 95% CI: 0.48–0.91), antiviral therapy (HR: 0.52; 95% CI: 0.43–0.62). In addition, the results of the Model A, Model B and Model C with adjustment for fewer variables were shown in Supplementary Table S5.

**Table 3:** Multivariate analysis for overall survival in patients with HCC.

	**HR**	***P***	**95%CI**
URRBMI (vs. UEBMI)	1.72	**<0.001**	1.47–2.00
Age (vs. 18–44)			
45–54	1.79	**0.002**	1.23–2.59
55–64	2.45	**<0.001**	1.72–3.49
65–74	3.43	**<0.001**	2.40–4.89
≥75	5.25	**<0.001**	3.66–7.53
Male (vs. female)	1.74	**<0.001**	1.51–2.00
CCI score	1.01	0.497	0.98–1.04
Compensated cirrhosis (vs. No)	1.34	**0.001**	1.12–1.60
Decompensated cirrhosis (vs. No)	1.85	**<0.001**	1.54–2.21
Hepatitis (vs. No)	0.92	0.314	0.78–1.08
Alcoholic liver disease (vs. No)	0.88	0.457	0.62–1.24
Fatty liver disease (vs. No)	0.66	**0.012**	0.48–0.91
Hepatic failure (vs. No)	0.99	0.890	0.81–1.19
Baseline total cost	1.00	0.561	1.00–1.00
Baseline ALOS	1.00	0.556	0.99–1.00
Any baseline outpatient visits (vs. No)	2.01	**<0.001**	1.72–2.34
Tumor metastasis at diagnosis (vs. No)	2.58	**<0.001**	2.19–3.04
Antiviral therapy during the follow-up (vs. No)	0.52	**<0.001**	0.43–0.62

*The Cox model was stratified by initial treatment after diagnosis and was broadly categorized as curative surgery (including hepatectomy and liver transplantation), non-curative surgery (including transarterial chemoembolization [TACE] and ablation), or no surgery. CCI score, Charlson Comorbidity Index score; ALOS, Average length of stay. Bold values means P < 0.05*.

### Lifetime Survival of HCC Patients

**Figure 3:**
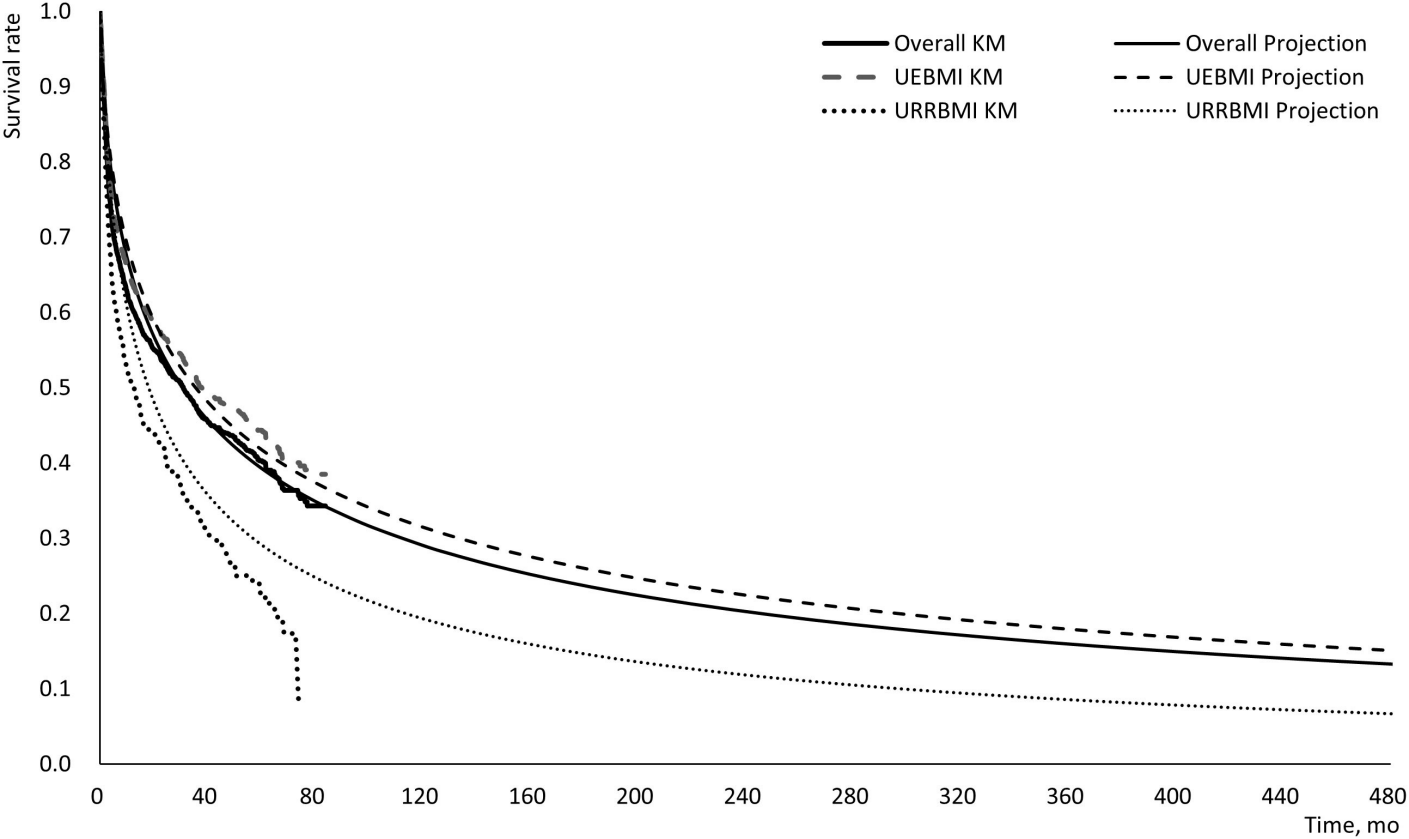
Log-normal projection survival curves for patients with HCC during the lifetime. UEBMI, Urban Employee Basic Medical Insurance; URRBMI, Urban and Rural Resident Basic Medical Insurance; KM, Kaplan-Meier; HCC, hepatocellular carcinoma.

Based on the total cohort's mean age (60.7 years old), overall survival curves were extrapolated to 40 years (i.e., 480 months) after the diagnosis of HCC to cover the lifetime horizon (Figure 3). The 10-year survival rates among the total cohort, the UEBMI cohort and the URRBMI cohort were 27.1, 31.5, and 13.1%, respectively. The results using Generalized gamma models did not vary significantly from those observed in the Log-normal models (Supplementary Figure S6).

### Sensitivity Analysis

The survival difference was reduced but still statistically significant (URRBMI vs. UEBMI, HR: 1.49; 95% CI: 1.21–1.83) in the two cohorts after propensity score matching. Baseline characteristics, Kaplan-Meier survival curves and multiple Cox proportional hazards models for the two cohorts after matching were reported in the supplementary (Supplementary Tables S3, S6, Supplementary Figure S6).

## Discussion

To the best of our knowledge, this is the first study to investigate the disparities in HCC survival by basic medical insurance in China as well as the first study to examine the discrepancies between UEBMI and URRBMI. In this population-based study, we found evidence of disparities in HCC survival by insurance status; the patients enrolled in URRBMI might have a higher risk of death than those enrolled in UEBMI whether during the follow-up period or over their lifetime.

Similar results were found in previous studies. In a study based on the data derived from Beijing Cancer Registry, underinsured (uninsured or NRCMS) patients with non-small cell lung cancer had shorter cancer-specific survival than well-insured (UEBMI or Free Medical Care) individuals (HR: 1.24; 95% CI: 1.03–1.49; *P* = 0.021) after adjusting for age, sex, cancer stage, smoking status, family history and residential area (22). Another study based on the Breast Cancer Information Management System in Sichuan West China Hospital has also suggested that patients covered by rural schemes (i.e., NRCMS) faced a higher risk of breast cancer-specific mortality (HR: 1.29; 95% CI: 1.00–1.65; *P* = 0.046) than those covered by urban schemes (URBMI, UEBMI, and/or commercial insurances) when adjusted for age, calendar year at diagnosis, ethnic group, education level, marital status, comorbidity, tumor characteristics (for example, histological type, hormone receptor status, tumor stage) and treatment (23). Compared with the previous studies, the HR of death for UEBMI and URRBMI cohorts in this study was larger (primary analysis: HR: 1.72; 95% CI: 1.47–2.00; sensitivity analysis HR: 1.49; 95% CI: 1.21–1.83). A possible reason might be that variables related to socioeconomic status (SES), such as educational level, income, and work status, were not sufficiently considered in this study. Enrollees in UEBMI always have a relatively higher SES and may pay close attention to health status, get more cancer screenings, and have full access to medical treatment. Additionally, some studies have also demonstrated that lower SES was associated with worse HCC-specific survival (30–32). Furthermore, previous studies examining the relationship between insurance and survival in other countries also have shown that patients with a good insurance status have better survival than those with poor insurance status, not only among HCC patients, but also among many other cancers (8–18).

Several mechanisms may contribute to the observed disparities in HCC survival between UEBMI and URRBMI. Firstly, patients with poor benefit packages are likely to have less access to healthcare (33). In this study, patients in the URRBMI cohort used fewer healthcare resources during the baseline period and had lower CCI scores. However, it did not mean that patients covered by URRBMI were in better health status, because they were found to be more likely to have some sorts of severe liver diseases including decompensated cirrhosis, liver failure and ascites during the baseline period. In addition, previous studies have reported that patients with inadequate insurance tended to receive cancer screening less frequently and were more likely to have an advanced stage of malignancy at diagnosis, which may be related to worse survival (22, 23, 34, 35). Even without the tumor stage variables in the database, this study showed that more patients in the URRBMI cohort had metastasized at diagnosis (Supplementary Table S7). In addition, when tumor metastasis at diagnosis and initial treatment after diagnosis were adjusted in the model, HR decreased from 2.14 (95% CI: 1.84–2.49, Model B) to 1.86 (95% CI: 1.59–2.16, Model C), which suggests that the survival disparity between UEBMI and URRBMI may exist before diagnosis (Supplementary Table S5). Secondly, the insurance status may also impact the treatment options, especially for uncovered therapies or with higher out-of-pocket expenses. Disparities in treatment by insurance status have been observed in the United States, with privately insured patients with HCC consistently being more likely to receive hepatectomy (35, 36). Some studies indicated that cancer patients with lower reimbursement rates were less likely to receive adjuvant chemotherapy and postoperative radiation therapy and were less likely to afford the high out-of-pocket expenses for an emerging therapy that significantly improved survival (e.g., targeted agents and immune agents) in China (23, 37). Herein, there were 11.7, 26.7, 61.6% and 11.6, 29.8, 58.6% patients with curative surgery, non-curative surgery, and no surgery for HCC patients in the UEBMI and the URRBMI cohorts, respectively (*P* = 0.356; Supplementary Table S7). There was no significant difference in receiving surgery between the two cohorts. Still, the preoperative and postoperative adjuvant therapy was not further analyzed due to insufficient power. Sorafenib was the only emerging drug approved for advanced HCC during the study period, but the basic medical insurance had not covered it by December 2017. Therefore, we could not examine whether more HCC patients enrolled in UEBMI had been treated with Sorafenib. In addition, the insurance status can be an indicator for health consciousness, health habits or socioeconomic status in this study, which might contribute to the survival (37, 38).

To understand the potential mechanisms contributing to the observed disparities in HCC survival, some additional analyses on the relationship between reimbursement rate (defined as the anti-cancer medical costs paid by basic medical insurance divided by the anti-cancer total costs in the insurance coverage) and HCC survival had been conducted. When the reimbursement rate was additionally adjusted in Model D, the HR of insurance type (URRBMI vs. UEBMI) decreased from 1.49 (95% CI: 1.21–1.83) to 1.42 (95% CI: 1.13–1.79) among matched cohorts (see Supplementary Table S8), which suggests that small part of the disparity in survival between UEBMI and URRBMI may be attributed to reimbursement rate. But further research is warranted to clarify the mechanisms by which health insurance affects survival.

Some factors also appeared to be associated with HCC survival in this study, consistent with previous studies. The risk of death increased with age, and patients who were 45 years old or older had a significantly higher risk of death than those younger than 45 years old. Males with HCC had worse survival than females, which was well-established in a previous study recruiting Americans (15, 39). Liver cirrhosis including compensated cirrhosis and decompensated cirrhosis were also related to the decreased survival, which was demonstrated among patients with HCC in Taiwan, China (40). Notably, some studies have indicated that hepatitis and liver cirrhosis are risk factors for HCC, and chronic hepatitis might lead to cirrhosis and then to HCC or other types of liver cancer. About 45% of patients had hepatitis (mainly HBV and HCV) or cirrhosis, respectively, before being diagnosed with HCC in this study. Therefore, regular screening and monitoring for patients with hepatitis or cirrhosis may contribute to the earlier diagnosis and better survival.

Antiviral therapy was also found to be associated with increased survival. To be mentioned, there were about 44.1 and 44.9% of patients in UEBMI and URRBMI cohorts with hepatitis during the baseline period, but the proportion of patients taking antiviral therapy were only 22.6 and 9.3% during the follow-up period (Supplementary Table S7). It is possible that some patients were cured during the baseline period. Still, HCC patients with hepatitis in the URRBMI cohort were less likely to receive antiviral treatment than those in the UEBMI cohort. Literature also reported that some antiviral regimens had better efficacy but were more expensive, and the benefits of these new antiviral regimens might not be accessible to all patients (41). Fatty liver disease was also associated with increased survival in the primary analysis (HR: 0.66; 95% CI: 0.48–0.91), but the association attenuated in the sensitivity analysis (HR: 0.54; 95% CI: 0.26–1.11). As fatty liver is a disease with no apparent clinical symptom, patients with URRBMI were more likely undiagnosed based on the discussion above. Therefore, the impact of fatty liver showed by the primary analysis might be biased. In addition, as there were no tumor stage variables in the database, we examined the tumor metastasis at diagnosis in the multiple Cox models, which was demonstrated to be associated with decreased HCC survival. Our findings highlight the importance of early screening and diagnosis for high-risk individuals.

Furthermore, this is also the first study to examine the survival of patients with HCC in mainland China. The median survival time was 31.0 months, which was similar to that of the Chinese patients in the U.S. (34.0 months) (42). The 1-, 3-, 5-, 10-year overall survival rates in this study were 60.6, 47.3, and 40.3%, respectively, which were slightly lower than in Taiwan, China (71.68, 57.14, and 47.82%) (40).

There are also some limitations to this study. Firstly, this study was conducted based on the Basic Medical Insurance claims database in Tianjin. The disparities in benefit packages by UEBMI and URRBMI may differ from those in other provinces. However, compared with UEBMI, patients enrolled in URRBMI continuously suffer from poorer benefit packages in almost all regions of China. Therefore, the results presented in this study, to a certain degree, could reflect the disparities in HCC survival by basic medical insurance in China. Secondly, this study did not examine the HCC-specific survival due to a lack of related information in the database. Nevertheless, studies that examined both cancer-specific survival and all-cause survival have reported similar results for the two outcome measures (15, 23). Thirdly, the database does not collect data on clinical characteristics (e.g., tumor stage), health behaviors (e.g., smoking, drinking), SES (e.g., education, income, work status) and private insurance. These factors likely differ between patients enrolled in UEBMI and URRBMI, especially SES and private insurance. If we were able to control for these factors, the HR of death for UEBMI and URRBMI cohorts in this study might decrease. Lastly, emerging therapies (i.e., Sorafenib) and some other prognostic factors (e.g., time to treatment, the preoperative and postoperative adjuvant therapy, complications related to the therapy and the treatment) related to treatment were not included, which might have an impact on HCC survival. Future studies using richer information on clinical characteristics, treatments, and SES are warranted to understand better the HCC survival disparities examined in this study.

## Conclusion

This study reveals that HCC patients covered by URRBMI may have worse survival than patients covered by UEBMI. Further efforts are warranted to understand healthcare disparities for patients covered by different basic medical insurance in China.

## Data Availability Statement

The data that support the findings of this study were available from the Tianjin Healthcare Security Administration. Due to the requirement from the data owner, these data could only be used for this study under the license, which could not be shared to others. Requests to access these datasets should be directed to Tianjin Healthcare Security Administration.

## Ethics Statement

The studies involving human participants were reviewed and approved by the Safety and Ethics Committee of the School of Pharmaceutical Science and Technology in Tianjin University. Written informed consent for participation was not required for this study in accordance with the national legislation and the institutional requirements.

## Author Contributions

JW and CL designed the study and supervised data collection. CL analyzed the data, interpreted the results, and drafted the manuscript. JW and FW critically reviewed the manuscript. All authors have read and agreed to the published version of the manuscript.

## Conflict of Interest

The authors declare that the research was conducted in the absence of any commercial or financial relationships that could be construed as a potential conflict of interest.

## Publisher's Note

All claims expressed in this article are solely those of the authors and do not necessarily represent those of their affiliated organizations, or those of the publisher, the editors and the reviewers. Any product that may be evaluated in this article, or claim that may be made by its manufacturer, is not guaranteed or endorsed by the publisher.
