# Supplementary material for: Disparities in Hepatocellular Carcinoma Survival by Insurance Status: A Population-Based Study in China
Source: Front Public Health. 2021 Nov 5;9:742355. doi: 10.3389/fpubh.2021.742355 (PMC8602862; doi:10.3389/fpubh.2021.742355)
Supplement: Supplementary file 1 [file Data_Sheet_1.docx]

***Supplementary material***

[Table S1. The ICD-10 codes used for the identification of liver comorbidities and complications 1](#_Toc82204125)

[Table S2. Goodness of fit statistics to different distributions 2](#_Toc82204126)

[Table S3. Baseline characteristics for patients with HCC after matching 3](#_Toc82204127)

[Table S4. Follow-up period for patients with HCC 4](#_Toc82204128)

[Table S5. Multivariate analysis for overall survival in patients with HCC 5](#_Toc82204129)

[Table S6. Multivariate analysis for overall survival in patients with HCC after matching 6](#_Toc82204130)

[Table S7. Initial treatment, antiviral therapy, and tumor metastasis for patients with HCC 7](#_Toc82204131)

[Table S8. Association of insurance status with overall survival when adjusted for reimbursement rate in patients with HCC after matching 8](#_Toc82204132)

[Figure S1. Projection survival curve for HCC patients enrolled in UEBMI. 9](#_Toc82204133)

[Figure S2. Projection survival curve for HCC patients enrolled in URRBMI. 9](#_Toc82204134)

[Figure S3. Projection survival curve for all HCC patients. 10](#_Toc82204135)

[Figure S4. Kaplan-Meier (KM) and Generalized gamma projection survival curves for patients with HCC during the lifetime. 10](#_Toc82204136)

[Figure S5. Kaplan-Meier survival curves during the follow-up period for patients with HCC in different subgroups. 11](#_Toc82204137)

[Figure S6. Kaplan-Meier survival curves during the follow-up period for HCC patients after matching. 12](#_Toc82204138)

| **Table S1. The ICD-10 codes used for the identification of liver comorbidities and complications** | |
| --- | --- |
|  | **ICD-10 codes** |
| Hepatitis | B16*,B18.0*,B18.1*,B17.1*,B18.2*,B15*,B17*,B18*,B19* ,K73* |
| HBV | B16*,B18.0*,B18.1* |
| HCV | B17.1*,B18.2* |
| Cirrhosis of liver | K74* |
| Hepatic failure | K72* |
| Fatty liver disease | K76.0 |
| Alcoholic liver disease ^‡^ | K70* |
| Ascites | R18* |
| Jaundice | R17* |
| Hepatorenal syndrome | K76.7 |
| Hepatic encephalopathy | Chinese descriptions |
| Portal hypertension | Chinese descriptions |
| Esophageal variceal bleeding | Chinese descriptions |
| Primary peritonitis | Chinese descriptions |

| **Table S2. Goodness of fit statistics to different distributions** | | | |
| --- | --- | --- | --- |
|  | **Log likelihood** | **AIC** | **BIC** |
| **Overall** |  |  |  |
| Weibull | -3,252.8 | 6,509.7 | 6,520.9 |
| Gompertz | -3,262.3 | 6,528.6 | 6,539.9 |
| Exponential | -3,591.7 | 7,185.3 | 7,190.9 |
| Log-normal ^†^ | **-3,174.7** | **6,353.4** | **6,364.6** |
| Log-logistic | -3,209.2 | 6,422.4 | 6,433.7 |
| Generalized gamma ^‡^ | **-3,146.7** | **6,299.4** | **6,316.3** |
| **UEBMI** |  |  |  |
| Weibull | -2,394.7 | 4,793.3 | 4,803.9 |
| Gompertz | -2,404.7 | 4,813.3 | 4,823.9 |
| Exponential | -2,637.0 | 5,276.0 | 5,281.3 |
| Lognomal ^†^ | **-2,342.4** | **4,688.8** | **4,699.4** |
| Loglogistic | -2,367.2 | 4,738.3 | 4,748.9 |
| Generalized gamma ^‡^ | **-2,322.7** | **4,651.4** | **4,667.3** |
| **URRBMI** |  |  |  |
| Weibull | -823.9 | 1,651.9 | 1,660.5 |
| Gompertz | -828.4 | 1,660.8 | 1,669.5 |
| Exponential | -885.9 | 1,773.8 | 1,778.2 |
| Lognomal ^†^ | **-803.1** | **1,610.2** | **1,618.9** |
| Loglogistic | -811.1 | 1,626.3 | 1,635.0 |
| Generalized gamma ^‡^ | **-800.1** | **1,606.2** | **1,619.2** |
| *Note.* The smaller AIC, smaller BIC and larger Log likelihood values were, the better it fitted. Although the Generalized gamma model generally provided the best AIC and BIC fit, it tended to generate long-tailed curve that might overestimate the survival curve. Log-normal was used in primary analysis, and Generalized gamma model, as the suboptimal distributions, was used for sensitivity analysis. | | | |

| **Table S3. Baseline characteristics for patients with HCC after matching** | | | |
| --- | --- | --- | --- |
|  | **UEBMI** | **URRBMI** | ***P*** |
|  | **(N=413)** | **(N=413)** |  |
| **Demographic characteristics** |  |  |  |
| Age [Mean(SD)] | 60.8(13.5) | 60.6(11.6) | 0.834 |
| Female [N(%)] | 126(30.5%) | 121(29.3%) | 0.704 |
| **Comorbidities and Complications [N(%)]** |  |  |  |
| CCI score [Mean(SD)] | 3.9(1.8) | 3.8(1.9) | 0.697 |
| Comorbidities related to liver |  |  |  |
| Hepatitis | 175(42.4%) | 166(40.2%) | 0.525 |
| HBV | 148(35.8%) | 139(33.7%) | 0.511 |
| HCV | 15(3.6%) | 8(1.9%) | 0.139 |
| Cirrhosis of liver | 194(47.0%) | 184(44.6%) | 0.161 |
| Compensated Cirrhosis | 110(26.6%) | 97(23.5%) | 0.297 |
| Decompensated Cirrhosis ^†^ | 84(20.3%) | 77(18.6%) | 0.539 |
| Hepatic failure | 54(13.1%) | 43(10.4%) | 0.234 |
| Fatty liver disease | 13(3.1%) | 10(2.4%) | 0.526 |
| Alcoholic liver disease ^‡^ | 5(1.2%) | 9(2.2%) | 0.281 |
| Ascites | 64(15.5%) | 62(15.0%) | 0.847 |
| Hepatic encephalopathy | 38(9.2%) | 33(8.0%) | 0.535 |
| Jaundice | 27(6.5%) | 25(6.1%) | 0.774 |
| Portal hypertension | 14(3.4%) | 14(3.4%) | 1.000 |
| Esophageal variceal bleeding | 13(3.1%) | 7(1.7%) | 0.174 |
| Primary peritonitis | 8(1.9%) | 8(1.9%) | 1.000 |
| Hepatorenal syndrome | 3(0.7%) | 3(0.7%) | 1.000 |
| **All-cause resource utilization and medical costs** |  |  |  |
| Total cost [Mean(SD, CNY] | 4,806(10,015) | 4,777(16,857) | 0.976 |
| CCI score, Charlson Comorbidity Index score; CNY, Chinese yuan (year-2017 1 USD = 6.77 CNY).  † Patients with liver cirrhosis who had the following symptoms was defined as decompensated liver cirrhosis: ascites; esophageal variceal bleeding; hepatorenal syndrome; portal hypertension; Hepatic encephalopathy and jaundice; hepatic encephalopathy and primary peritonitis; jaundice and primary peritonitis.  ‡ Including alcoholic liver cirrhosis, alcoholic hepatitis, alcoholic fatty liver disease and alcoholic liver failure; the hepatitis, liver cirrhosis, fatty liver disease and liver failure in this table only included non-alcoholic disease. | | | |

| **Table S4. Follow-up period for patients with HCC** | | | | | | | | | |
| --- | --- | --- | --- | --- | --- | --- | --- | --- | --- |
|  | **Overall N=2,068** | | | **UEBMI N=1,498** | | | **URRBMI N=570** | | |
|  |  | **Follow up period, mo** | |  | **Follow up period, mo** | |  | **Follow up period, mo** | |
|  | **N(%)** | **mean** | **meidan** | **N(%)** | **mean** | **meidan** | **N(%)** | **mean** | **meidan** |
| No. of subjects | 2,068(100.0%) | 24.6 | 14.8 | 1,498(100.0%) | 29.2 | 22.5 | 570(100.0%) | 12.6 | 4.6 |
| Reasons for the end of follow-up | |  |  |  |  |  |  |  |  |
| Death due to any cause | 1,085(52.5%) | 11.6 | 4.3 | 783(52.3%) | 12.6 | 4.8 | 302(53.0%) | 8.8 | 3.2 |
| Withdrawal from the medical insurance | 464(22.4%) | 27.6 | 22.1 | 229(15.3%) | 42.1 | 43.3 | 235(41.2%) | 13.5 | 6.9 |
| Follow-up to the latest data in the dataset | 519(25.1%) | 49.3 | 49.1 | 486(32.4%) | 49.7 | 50.3 | 33(5.8%) | 40.6 | 40.7 |

| **Table S5. Multivariate analyses for overall survival in patients with HCC before matching** | | | | | | | | |
| --- | --- | --- | --- | --- | --- | --- | --- | --- |
|  | **Model A** | | **Model B** | | **Model C** | | **Model D** | |
|  | **HR (95% CI)** | ***P*** | **HR (95% CI)** | ***P*** | **HR (95% CI)** | ***P*** | **HR (95% CI)** | ***P*** |
| URRBMI (vs. UEBMI) | 2.17 (1.88-2.51) | **<0.001** | 2.14 (1.84-2.49) | **<0.001** | 1.86 (1.59-2.16) | **<0.001** | 1.72 (1.47-2.00) | **<0.001** |
| Age (vs. 18-44) |  |  |  |  |  |  |  |  |
| 45-54 | 1.84 (1.28-2.66) | **0.001** | 1.73 (1.20-2.51) | **0.003** | 1.75 (1.21-2.53) | **0.003** | 1.79 (1.23-2.59) | **0.002** |
| 55-64 | 2.61 (1.84-3.7) | **<0.001** | 2.41 (1.70-3.43) | **<0.001** | 2.39 (1.68-3.41) | **<0.001** | 2.45 (1.72-3.49) | **<0.001** |
| 65-74 | 3.95 (2.77-5.61) | **<0.001** | 3.64 (2.56-5.20) | **<0.001** | 3.52 (2.47-5.03) | **<0.001** | 3.43 (2.40-4.89) | **<0.001** |
| ≥75 | 6.54 (4.58-9.34) | **<0.001** | 6.13 (4.28-8.79) | **<0.001** | 5.46 (3.81-7.82) | **<0.001** | 5.25 (3.66-7.53) | **<0.001** |
| Male (vs. female) | 0.62 (0.54-0.71) | **<0.001** | 0.62 (0.54-0.71) | **<0.001** | 0.58 (0.51-0.67) | **<0.001** | 0.58 (0.50-0.66) | **<0.001** |
| Baseline total cost | 1.00 (1.00-1.00) | 0.636 | 1.00 (1.00-1.01) | 0.328 | 1.00 (1.00-1.00) | 0.492 | 1.00 (1.00-1.00) | 0.636 |
| Baseline ALOS | 1.00 (1.00-1.01) | 0.748 | 1.00 (0.99-1.01) | 0.885 | 1.00 (0.99-1.01) | 0.852 | 1.00 (0.99-1.00) | 0.748 |
| Any baseline outpatient visits (vs. No) | 2.13 (1.84-2.46) | **<0.001** | 2.07 (1.79-2.41) | **<0.001** | 1.95 (1.67-2.28) | **<0.001** | 2.01 (1.72-2.34) | **<0.001** |
| CCI score | - | - | 1.03 (0.99-1.06) | 0.116 | 1.02 (0.99-1.05) | 0.289 | 1.01 (0.98-1.04) | 0.561 |
| Compensated Cirrhosis (vs. No) | - | - | 1.24 (1.04-1.48) | **0.015** | 1.30 (1.09-1.55) | **0.004** | 1.34 (1.12-1.60) | 0.556 |
| Decompensated Cirrhosis (vs. No) | - | - | 1.92 (1.61-2.30) | **<0.001** | 1.81 (1.51-2.17) | **<0.001** | 1.85 (1.54-2.21) | **<0.001** |
| Hepatitis (vs. No) | - | - | 0.76 (0.65-0.88) | **<0.001** | 0.77 (0.66-0.90) | **0.001** | 0.92 (0.78-1.08) | 0.497 |
| Alcoholic liver disease (vs. No) | - | - | 0.99 (0.70-1.40) | 0.957 | 0.94 (0.66-1.33) | 0.709 | 0.88 (0.62-1.24) | **0.001** |
| Fatty liver disease (vs. No) | - | - | 0.66 (0.48-0.92) | **0.012** | 0.68 (0.49-0.94) | **0.018** | 0.66 (0.48-0.91) | **<0.001** |
| Hepatic failure (vs. No) | - | - | 0.90 (0.74-1.08) | 0.253 | 0.96 (0.79-1.16) | 0.648 | 0.99 (0.81-1.19) | 0.314 |
| Tumor metastasis at diagnosis (vs. No) | - | - | - | - | 2.68 (2.27-3.16) | **<0.001** | 2.58 (2.19-3.04) | 0.457 |
| Antiviral therapy during follow-up (vs. No) | - | - | - | - | - | - | 0.52 (0.43-0.62) | **0.012** |
| *Note.* Model C and Model D were stratified by initial treatment after diagnosis and was broadly categorized as curative surgery (including hepatectomy and liver transplantation), non-curative surgery (including transarterial chemoembolization [TACE] and ablation), or no surgery.  CCI score, Charlson Comorbidity Index score; ALOS, Average length of stay. | | | | | | | | |

| **Table S6. Multivariate analysis for overall survival in patients with HCC after matching** | | | |
| --- | --- | --- | --- |
|  | **HR** | ***P*** | **95%CI** |
| URRBMI (vs. UEBMI) | 1.49 | **<0.001** | 1.21-1.83 |
| Age (vs. 18-44) |  |  |  |
| 45-54 | 1.83 | 0.057 | 0.98-3.42 |
| 55-64 | 3.45 | **<0.001** | 1.90-6.24 |
| 65-74 | 4.01 | **<0.001** | 2.19-7.31 |
| ≥75 | 5.90 | **<0.001** | 3.22-10.79 |
| Male (vs. female) | 1.72 | **<0.001** | 1.38-2.15 |
| CCI score | 1.05 | 0.158 | 0.98-1.11 |
| Compensated Cirrhosis (vs. No) | 1.56 | **0.001** | 1.19-2.05 |
| Decompensated Cirrhosis (vs. No) | 1.57 | **0.003** | 1.17-2.12 |
| Hepatitis (vs. No) | 0.82 | 0.131 | 0.64-1.06 |
| Alcoholic liver disease (vs. No) | 0.54 | 0.084 | 0.27-1.08 |
| Fatty liver disease (vs. No) | 0.54 | 0.092 | 0.26-1.11 |
| Hepatic failure (vs. No) | 1.14 | 0.426 | 0.82-1.59 |
| Baseline total cost | 1.00 | 0.855 | 1.00-1.00 |
| Baseline ALOS | 0.99 | 0.466 | 0.98-1.01 |
| Any baseline outpatient visits (vs. No) | 1.52 | **<0.001** | 1.23-1.89 |
| Tumor metastasis at diagnosis (vs. No) | 2.55 | **<0.001** | 1.97-3.31 |
| Antiviral therapy during follow-up (vs. No) | 0.53 | **<0.001** | 0.39-0.71 |
| *Note.* This Cox model was stratified by initial treatment after diagnosis and was broadly categorized as curative surgery (including hepatectomy and liver transplantation), non-curative surgery (including transarterial chemoembolization [TACE] and ablation), or no surgery.  CCI score, Charlson Comorbidity Index score; ALOS, Average length of stay. | | | |

| **Table S7. Initial treatment, antiviral therapy, and tumor metastasis for patients with HCC** | | | | |
| --- | --- | --- | --- | --- |
|  | **Overall** | **UEBMI** | **URRBMI** | **P值** |
| [N(%)] | **(N=2,068)** | **(N=1,498)** | **(N=570)** |  |
| **Initial treatment** |  | | | 0.356 |
| Curative surgery | 242(11.7%) | 176(11.7%) | 66(11.6%) |  |
| Non-curative surgery | 570(26.7%) | 400(26.7%) | 170(29.8%) |  |
| No surgery | 1,256(61.6%) | 922(61.6%) | 334(58.6%) |  |
| **Antiviral therapy** | 391(18.9%) | 338(22.6%) | 53(9.3%) | **<0.001** |
| **Tumor metastasis at diagnosis** | 240(11.6%) | 148(9.9%) | 92(16.1%) | **<0.001** |
| *Note.* The initial treatment was broadly categorized as curative surgery (including hepatectomy and liver transplantation), non-curative surgery (including transarterial chemoembolization [TACE] and ablation), or no surgery (without any surgery above mentioned). Only patients had twice prescriptions of antiviral medication during the follow-up period was defined as receiving antiviral therapy | | | | |

| **Table S8. Association of insurance status with overall survival when adjusted for reimbursement rate in patients with HCC after matching** | | | |
| --- | --- | --- | --- |
|  | **HR** | ***P*** | **95%CI** |
| URRBMI (vs. UEBMI) | 1.42 | **0.003** | 1.13-1.79 |
| Reimbursement rate |  |  |  |
| 0%-50% (vs. 51%-100%) | 1.12 | 0.368 | 0.87-1.44 |
| Age (vs. 18-44) |  |  |  |
| 45-54 | 1.85 | **0.054** | 0.99-3.44 |
| 55-64 | 3.53 | **<0.001** | 1.94-6.40 |
| 65-74 | 4.15 | **<0.001** | 2.26-7.60 |
| ≥75 | 6.13 | **<0.001** | 3.33-11.28 |
| Male (vs. female) | 0.58 | **<0.001** | 0.46-0.72 |
| CCI score | 1.05 | 0.134 | 0.99-1.12 |
| Compensated Cirrhosis (vs. No) | 1.56 | **0.001** | 1.19-2.05 |
| Decompensated Cirrhosis (vs. No) | 1.58 | **0.002** | 1.17-2.14 |
| Hepatitis (vs. No) | 0.81 | 0.112 | 0.63-1.05 |
| Alcoholic liver disease (vs. No) | 0.54 | 0.082 | 0.27-1.08 |
| Fatty liver disease (vs. No) | 0.53 | 0.086 | 0.26-1.09 |
| Hepatic failure (vs. No) | 1.14 | 0.424 | 0.82-1.59 |
| Baseline total cost | 1.00 | 0.912 | 1.00-1.00 |
| Baseline ALOS | 0.99 | 0.436 | 0.98-1.01 |
| Any baseline outpatient visits (vs. No) | 1.54 | **<0.001** | 1.24-1.90 |
| Tumor metastasis at diagnosis (vs. No) | 2.57 | **<0.001** | 1.99-3.34 |
| Antiviral therapy during follow-up (vs. No) | 0.53 | **<0.001** | 0.39-0.72 |
| *Note.* The reimbursement rate was defined as the amount of anti-cancer medical costs paid by basic medical insurance divided by the anti-cancer total costs in the insurance coverage, of which the median was about 51% in this study, and the average reimbursement rate of UEBMI was twenty percentage points higher than that of URRBMI.  This Cox model was stratified by initial treatment after diagnosis and was broadly categorized as curative surgery (including hepatectomy and liver transplantation), non-curative surgery (including transarterial chemoembolization [TACE] and ablation), or no surgery.  CCI score, Charlson Comorbidity Index score; ALOS, Average length of stay. | | | |

# **Figure S1. Projection survival curve for HCC patients enrolled in UEBMI.**

# **Figure S2. Projection survival curve for HCC patients enrolled in URRBMI.**

# **Figure S3. Projection survival curve for all HCC patients.**

# **Figure S4. Kaplan-Meier (KM) and Generalized gamma projection survival curves for patients with HCC during the lifetime.**

# **Figure S5. Kaplan-Meier survival curves during the follow-up period for patients with HCC in different subgroups.**

A. For HCC patients in different age subgroups

B. For HCC patients in different sex subgroups

C. For HCC patients in different Charlson Comorbidity Index (CCI) score subgroups

D. For HCC patients in different cirrhosis subgroups

E. For HCC patients in different hepatitis subgroups

# **Figure S6. Kaplan-Meier survival curves during the follow-up period for HCC patients after matching.**
